# Editorial: Dismantling disparities: advancing mental healthcare access for diverse youth

**DOI:** 10.3389/frcha.2025.1758566

**Published:** 2026-01-23

**Authors:** Miya L. Barnett, Jason F. Jent, Dainelys Garcia

**Affiliations:** 1 Department of Counseling, Clinical, and School Psychology, University of California, Santa Barbara, CA, United States; 2 Department of Pediatrics, Miller School of Medicine, University of Miami, Miami, FL, United States

**Keywords:** children's mental health care, culturally acceptable mental health treatments, mental health disparities for children and youth, mental health service access, mental health workforce

Disparities in access to, utilization, and effectiveness of mental healthcare for minoritized youth remains a persistent and urgent issue to address in pediatric care (1). Youth and families from racial, ethnic, sexual, and gender minority groups, along with those in rural settings, face a number of structural obstacles to accessing and receiving mental health services, including limited insurance coverage, provider shortages, fragmented systems of care, and a lack of culturally and linguistically responsive services. Additionally, experiences of discrimination, such as racism, transphobia, and sexism impact both a child's mental health, and the effectiveness of the services they receive (2).

This Research Topic, *Dismantling Disparities: Advancing Mental Healthcare Access for Diverse Youth*, was conceived to accelerate the behavioral health field's shift from identifying barriers to developing, implementing, and evaluating solutions. Although decades of research have documented significant access gaps for historically underserved children and youth, far less applied work has focused on innovative strategies intentionally designed to promote equity. The seven contributions in this Research Topic highlight important themes needed to address children's mental health disparities. Overall, achieving equitable access requires interventions that are responsive to the lived experiences, cultural identities, and contextual stressors of minoritized youth and families. Innovations across the entire care continuum are featured in this collection, from screening and referral, to workforce development, and the creation of culturally grounded therapeutic models.

As a first step to reach more families, Peskin et al. identify how to enhance the reach of evidence-based treatment by evaluating and iterating on referral strategies within a Parent-Child Interaction Therapy (PCIT) Clinic. Their study demonstrates that relatively simple procedural modifications, such as publicly accessible online screening forms, can significantly increase families' likelihood of completing the initial steps toward care. Although these strategies alone did not fully resolve challenges related to intake attendance or treatment retention, the findings underscore the importance of optimizing logistical processes that often determine whether families even make it to the clinic door to start services.

In the domain of engagement within early childhood behavioral intervention, Cafatti Mac-Niven et al. offer novel insights into the role of cultural identity by examining how cultural identity shapes engagement in Internet-delivered PCIT for families of children with developmental delays. Their finding that caregivers’ continued connection to their cultural heritage predicts higher homework engagement points to the importance of identifying and promoting cultural strengths in treatment. This may be especially helpful within telehealth modalities, which continue to expand access for underserved families.

Extending the importance of integrating cultural values within behavioral health services, Mena et al. present enhancements to Culturally Informed and Flexible Family-Based Treatment for Adolescents designed to better address self-harm behaviors among diverse youth (Black, Latine, and LGBTQ+). Through a clinical case example, they illustrate how interventions can maintain fidelity while integrating cultural values, contextual stressors, and family processes that influence treatment engagement.

Moving beyond treatment development, multiple papers in this collection highlight the needs of therapists being trained in evidence-based practices. Chavez et al. examine how clinicians' training experiences shape their ability to deliver culturally congruent PCIT services for Black families.Through structured interviews with a racially diverse group of PCIT clinicians (Black, White, Asian, multiracial), the authors found broad agreement that while PCIT training is high-quality, it is largely presented through White cultural norms and offers limited guidance for working with Black families. Clinicians noted that Black families often disengaged from treatment earlier, emphasized the need for more Black PCIT providers, and reported varying levels of confidence in knowing how to address the unique needs of Black families with PCIT. These insights highlight opportunities to strengthen PCIT training by more intentionally integrating cultural responsiveness and supporting a more diverse clinician workforce.

Relatedly, Onovbiona et al., present a qualitative analysis of a culturally adapted PCIT training program for Black and Latine clinicians serving autistic youth. Their findings reveal that training environments designed to be culturally supportive, including but not limited to incorporating shared cultural identities, attention to systemic barriers, and community-building among trainees, can strengthen clinicians' sense of competence, reduce isolation, and improve the dissemination of evidence-based care to families historically underserved or misdiagnosed by mainstream systems.

Given the national shortages of licensed mental health clinicians to meet the demands for services, innovative workforce expansions are equally essential. In their scoping review, Werntz et al. examine the emerging practice of therapeutic mentoring, a paraprofessional model with increasing relevance amid provider shortages and growing youth mental health needs. Their synthesis reveals both the promise and the limitations of this approach. Although therapeutic mentoring has been applied with diverse youth in community settings, the review identifies substantial gaps, including inconsistent definitions, limited rigorous evaluation, and a need for clearer training standards. Nevertheless, the model's potential for scalability and cultural alignment positions it as a promising strategy for extending the reach of mental health supports in underserved communities when thoughtfully developed.

Additionally, innovative models of care, such as integrated healthcare, are needed to address mental health inequities. Williford et al. mixed-methods study provides a rare window into how pediatric subspecialty providers in rural Appalachia experience the mental and behavioral health needs of medically complex youth. Their work highlights a persistent mismatch between the high prevalence of mental and behavioral health concerns among medically complex youth and the limited integration of mental health services within pediatric specialty settings. Notably, the authors document substantial readiness for change among providers and administrators, revealing an important lever for future systems-level improvement in rural and resource-limited settings.

Overall, this research collection underscores the multidimensional nature of access disparities and the equally multifaceted strategies needed to dismantle them. The articles collectively demonstrate that innovation must occur simultaneously across healthcare systems, clinical models, engagement processes, and workforce training. They also highlight that culturally responsive care is not an optional enhancement but a foundational component of effective intervention for minoritized youth.
